# Overexpression of DBT suppresses the aggressiveness of renal clear cell carcinoma and correlates with immune infiltration

**DOI:** 10.3389/fimmu.2023.1197011

**Published:** 2023-06-13

**Authors:** Chiyu Zhang, Gaomin Huang, Jiale Yang, Yi Jiang, Ruizhen Huang, Zhenfeng Ye, Yawei Huang, Honglin Hu, Xiaoqing Xi

**Affiliations:** ^1^ Department of Urology, The Second Affiliated Hospital of Nanchang University, Nanchang, China; ^2^ Hepato-Biliary-Pancreatic Surgery Division, Department of General Surgery, The Second Affiliated Hospital of Nanchang University, Nanchang, China

**Keywords:** DBT, prognostic model, tumor microenvironment, drug sensitivity, kidney renal clear cell carcinoma

## Abstract

Conventional therapy for kidney renal clear cell carcinoma (KIRC) is unpromising. The tumor microenvironment (TME) is intimately linked to the invasiveness of a variety of tumor forms, including KIRC. The purpose of this research is to establish the prognostic and immune-related significance of dihydrolipoamide branched chain transacylase E2 (DBT) in individuals with KIRC. In this investigation, we discovered that DBT expression was down-regulated in a range of human malignancies, and low DBT expression in KIRC was linked to higher-level clinicopathological characteristics as well as a poor prognosis for KIRC patients. Based on the findings of univariate and multivariate Cox regression analyses, DBT might be employed as an independent prognostic factor in KIRC patients. Furthermore, we developed a nomogram to better investigate DBT’s predictive usefulness. To confirm DBT expression, we examined KIRC cell lines using RT-qPCR and Western blotting. We also examined the role of DBT in KIRC using colony formation, CCK-8, EdU, transwell, and wound healing assays. We discovered that plasmid-mediated overexpression of DBT in KIRC cells slowed cell proliferation and decreased migration and invasion. Multiple enrichment analyses revealed that DBT may be involved in processes and pathways related to immunotherapy and drug metabolism. We computed the immune infiltration score and discovered that the immunological score and the ESTIMATE score were both greater in the DBT low expression group. According to the CIBERSORT algorithm, DBT seems to promote anti-cancer immune responses in KIRC by activating M1 macrophages, mast cells, and dendritic cells while inhibiting regulatory T cells. Finally, in KIRC, DBT expression was found to be highly linked to immunological checkpoints, targeted medicines, and immunotherapeutic agents. Our findings suggest that DBT is a distinct predictive biomarker for KIRC patients, playing a significant role in the TME of KIRC and serving as a reference for the selection of targeted treatment and immunotherapy.

## Introduction

Kidney renal clear cell carcinoma (KIRC) is one of the most aggressive urological tumors, exhibiting high resistance to chemotherapy and radiation (1). Obesity and smoking are both risk factors for KIRC, and they are associated with oxidative stress, which suggests their involvement in the development and maintenance of KIRC (2). Surgical resection is the preferred treatment for localized KIRC, but early diagnosis is rare, and the neoplasia often presents late in the disease process or with metastasis already occurring (3). Systemic therapies are available for more advanced stages of KIRC, including cytokine therapy or tyrosine kinase inhibitors combined with immunotherapy. However, these approaches are still inadequate (4). The branched-chain-keto acid dehydrogenase complex (BCKD) is an intramitochondrial enzyme complex that catalyzes the breakdown of branched-chain amino acids (BCAAs) (5). Dihydrolipoamide branched chain transacylase E2 (DBT) encodes one of the three subunits of BCKD, and mutations in this gene cause type 2 maple syrup urine disease (6).

Tumor tissue is made up of a variety of cellular components, including immune cells and stromal cells (7). A growing body of evidence suggests that tumor microenvironment (TME) is involved in a variety of biological behaviors of cancer cells, becoming a source of interest for researchers (8). It is an extremely complex mechanism behind how TMEs are formed. As a significant element of TME, tumor-infiltrating immune cells (TIICs) account for roughly 30 percent of the tumor mass and are regarded to play a key role in the carcinogenesis and development of a variety of cancers (9). Macrophages that are activated in the classical manner (M1) promote an anti-tumor response, whereas tumor-associated macrophages (TAMs) suppress the immune system (10). Through preventing T cell proliferation, promoting T cell apoptosis, and inhibiting cytotoxic T cell responses, TAMs suppress immune responses and form immunosuppressive microenvironments (11). According to many studies, a number of major mediators of inflammation contribute to the growth of tumors (12). The major leukocyte component, macrophages, plays a central role in the immune system of the host (13).

In this study, we evaluated the predictive value of DBT in pan-cancer patients and found that it has exceptional prognostic value in KIRC patients, prompting us to conduct further research. We performed extensive bioinformatics analysis to investigate the association between DBT and various clinicopathological features in individuals with KIRC. Based on the results of univariate and multivariate Cox regression analyses, we determined that DBT can be used as an independent prognostic factor and an independent risk factor in KIRC patients. Subsequently, we developed a nomogram to better assess the predictive utility of DBT. Our multiple enrichment analyses revealed that DBT may be involved in processes and pathways related to immunotherapy and drug metabolism. We also calculated TME-related scores for patients with different levels of DBT expression and examined their relationship with DBT. Additionally, we further investigated the association between DBT and immune infiltrating cells. To confirm DBT expression, we examined KIRC cell lines using RT-qPCR and Western blotting. We also conducted colony formation, CCK-8, EdU, transwell, and wound healing assays to examine the role of DBT in KIRC. Finally, we studied the relationship between DBT and immune checkpoint or medication sensitivity to gain a better understanding of the potential impact of immunotherapy on patients with KIRC.

## Materials and methods

### Acquisition and preprocessing of raw data

The pan-cancer DBT expression data was taken from the Tumor Immune Estimation Resource (TIMER, https://cistrome.shinyapps.io/timer/) (14). The Cancer Genome Atlas (TCGA) database was used to retrieve gene expression data (FPKM) and associated clinical characteristics (https://portal.gdc.cancer.gov/) (15). Subsequently, we converted RNA sequencing data from FPKM to TPM format, kept clinical data and RNA sequencing data, and evaluated all of the data according to the TCGA publishing rules. Because the data for this investigation was obtained from public databases such as TCGA and GEO, informed permission and ethical clearance were not necessary. Additionally, this work adhered to all applicable database publishing requirements.

### Assessment of diagnostic performance

The R package “limma” was used to analyze the differential expression of DBT among KIRC patients based on seven clinicopathological features, including age, gender, WHO grade, pathological stage, T stage, N stage, and M stage (16). In this survival analysis, the R packages “survival” and “survminer” were used. Furthermore, the ROC curves were plotted using “timeROC” to predict the 1-, 3-, and 5-year OS of KIRC patients in the TCGA dataset. Through analyzing the areas under the ROC curves (AUCs), we were able to compare the diagnostic performance of DBT. With the R package “survival”, six clinicopathological characteristics and DBT have been analyzed by univariate Cox regression. A multivariate Cox regression analysis was performed in order to determine if DBT expression is an independent prognostic factor for KIRC. Using the “rms” R package, a nomogram was created based on the independently predictive factors gleaned from multivariate Cox analysis. For verification of the nomogram’s accuracy, we generated calibration plots with the R packages “rms,” “regplot,” and “survival” in the TCGA dataset.

### Co-expression of DBT with other genes in KIRC

The genes co-expressed with DBT in the TCGA-KIRC dataset were identified using R. To determine the association between DBT and co-expressed genes, Spearman’s correlation coefficient was computed, with |r| > 0.60 and P < 0.001 being chosen. We then used the “limma”, “circlize”, and “corrplot” packages to obtain the 12 most correlated genes and depict the correlations with different colors and arrow sizes in the circle plots.

### Functional enrichment analysis

Using R package “limma”, we identified differentially expressed genes (DEGs, |log2 fold change (FC)| >1 and an adjusted P value <0.05) that differ between patients with high and low expression of DBT in KIRC. In order to determine which genes are associated with DBT expression, a Spearman correlation analysis was conducted. We used the “pheatmap” program to construct heat maps of the top 100 genes related to DBT. On the basis of DEGs, GO and KEGG enrichment analyses were conducted with the R packages “clusterProfiler”, “org.Hs.eg.db”, “enrichplot” and “ggplot2”. Following that, we used a gene set enrichment analysis (GSEA) to identify signaling pathways that differed between the DBT-high and -low groups. An enriched gene is considered significant if it has an adjusted P value of <0.05 and a false discovery rate (FDR) of <0.25. We use “limma”, “org.Hs.eg.db”, “clusterProfiler” and “enrichplot” R packages for GSEA analysis and show the top 5 pathways according to their relevance.

### Correlation analysis of immune cell infiltration

Each sample in the TCGA cohort was evaluated using the ESTIMATE algorithm to determine its immune score, stromal score, and ESTIMATE score, and then the results were visualized in a violin plot *via* the “limma”, “estimate”, “reshape2”, and “ggpubr” packages. We uploaded the gene expression matrix data to CIBERPORT, a tool for estimating the composition and number of immune cells in mixed cell populations, and established a p-value of 0.05 as a screening requirement to assess the immune cell infiltration matrix. To highlight disparities in the expression of specific immune cells in distinct DBT expression groups, histograms were displayed using the “ggpubr”, “vioplot”, and “ggExtra” software programs. Next, we plotted the correlation bar graphs using the R software to better show the correlation of various immune cell infiltrations. Additionally, we examined the relationship between DBT and multiple immune checkpoints using “limma”, “Reshape2”, “GgPlot2”, “Ggpubr”, and “Corrplot” packages, and presented the correlation as two heat maps.

### Drug sensitivity and immunotherapy analysis

Using the “pRRophetic” package, we calculated the semi-inhibitory concentrations (IC50) of chemotherapeutic agents commonly used to treat KIRC in order to investigate the differences in therapeutic effects among patients with different DBT expressions (17). Data for KIRC-IPS were retrieved from TCIA (http://tcia.at/home), a public database of cancer immunomics (18). According to the Immune Atlas, which provides information on cancer patients receiving immunotherapy, immunophenscore (IPS) was calculated on a scale of 0–10 based on gene expression data. We used the “limma”, “ggpubr” program packages to extract data from immunotherapy and present them in four violin plots depending on the treatment regimen.

### Cell culture and transfection

All cell lines were obtained from the Cell Bank of the Chinese Academy of Sciences (Shanghai, China). ACHN, A498, and HK-2 cell lines were cultured in MEM medium, while 786-O and 769-P cell lines were cultured in RPMI1640 medium (Gibco, NY, USA). The medium was supplemented with 10% fetal bovine serum (FBS; Gibco, NY, USA), 100 μg/ml streptomycin, and 100 U/ml sodium penicillin (Biotechnology, Beijing, China). Cells were cultured at 37°C in a humidified atmosphere containing 5% CO_2_. The control overexpression plasmid (Vector) and the DBT overexpression plasmid (OE-DBT) were constructed using the pcDNA 3.1(+) vector (Hanheng Biotechnology, Shanghai, China). A498 cells were transfected with the OE-DBT and Vector plasmids using Lipofectamine 3000 transfection reagent (Invitrogen, Waltham, Massachusetts, USA), following the manufacturer’s instructions.

### Quantitative real-time PCR and western blot

The Trizol technique was used to extract total RNA from tissues and cells. The cDNA (TaKaRa, RR047A) was then used for real-time quantitative PCR (TAKARA, RR420A). Data analysis was performed using the 2−ΔΔCt method. The real-time PCR primers used were as follows: for DBT, Forward Primer 5’-CAGTTCGCCGTCTGGCAAT-3’, Reverse Primer 5’-CCTGTGAATACCGGAGGTTTTG-3’; for PD-L1, Forward Primer 5′-TCACTTGGTAATTCTGGGAGC-3′, Reverse Primer 5′-CTTTGAGTTTGTATCTTGGATGCC-3′; for GAPDH, Forward Primer 5′-GGAGCGAGATCCCTCCAAAAT-3′, Reverse Primer 5′-GGCTGTTGTCATACTTCTCATGG-3′. GAPDH was used as the reference control. Western blotting was performed on total cell protein using primary antibodies against DBT (1:1000, Proteintech, Wuhan, China), PD-L1 (1:1000, Proteintech, Wuhan, China), and GAPDH (1:5000, Proteintech, Wuhan, China).

### Cell counting kit-8 assay

Utilizing a Cell Counting Kit-8 (CCK-8) we assessed cell proliferation (Bioss, Beijing, China). Transfected KIRC cells were seeded at a density of 2000 cells per well in 96-well plates and grown at 37 degrees Celsius and 5% carbon dioxide. At 0, 24, 48, and 72 hours, the CCK-8 reagent was added, and the samples were incubated for 2 hours. At 450 nm, the absorbance of each sample was measured using a microplate reader (Infinite 2000 PRO, TECAN, Switzerland).

### 5-ethynyl-2′-deoxyuridine (EdU) staining assays

Three thousand cells were seeded in 100 μl of complete media in each well of a 96-well plate and cultured overnight. Afterward, the cells were incubated for 3 hours in a medium containing 20 μM EdU. Following fixation with 4% paraformaldehyde, the cells were treated with 100 μl of Apollo dye solution (RiboBIO, Guangzhou, China) for 30 minutes. The cells were then washed for 10 minutes with 0.5% TritonX-100 and stained with DAPI. The number of EdU-positive cells was subsequently determined using ImageJ software.

### Colony formation assays

KIRC cells stably transfected with the gene of interest were seeded at a density of 800 cells per well onto 6-well plates and cultured at 37°C and 5% CO_2_ for two weeks. The cells were fixed with 4% paraformaldehyde for 30 minutes, stained with a 1% crystal violet solution at room temperature for 30 minutes, and washed three times with PBS. The number of clones with more than 50 cells per colony was counted using an inverted phase contrast microscope.

### Cell migration and invasion assays

The migratory and invasive abilities of cells were evaluated using chambers with an 8 μm pore size. The cells were suspended in serum-free media and added to the upper compartment (2×10^4^ cells) for the migration test. The bottom chamber contained 600 μl of MEM medium with 10% bovine fetal serum. Matrigel (BD Biosciences, United States) was pre-coated on the upper chamber for the invasion experiment. After 24 hours of incubation, the cells were fixed with 4% paraformaldehyde for 30 minutes and stained with 1% crystal violet for 30 minutes. The top surface of the chamber was lightly cleaned to remove any non-migratory cells. The migrating cells were counted and photographed using a phase-contrast microscope after fixation and staining.

### Wound healing assay

To measure cell migration, wound healing assays were used. Initially, the transfected KIRC cells were planted equally onto 6-well plates and cultivated until they achieved complete confluence. The cell monolayer was then softly scraped with a 200-μl sterile pipette tip. After washing the cells with PBS, serum-free media was added. At 0 and 24 hours after scratching cells, cell migration at the same place was examined under a microscope, and the scratch area was assessed using ImageJ.

## Results

### DBT expression is downregulated in KIRC tissue

**Figure 1:**
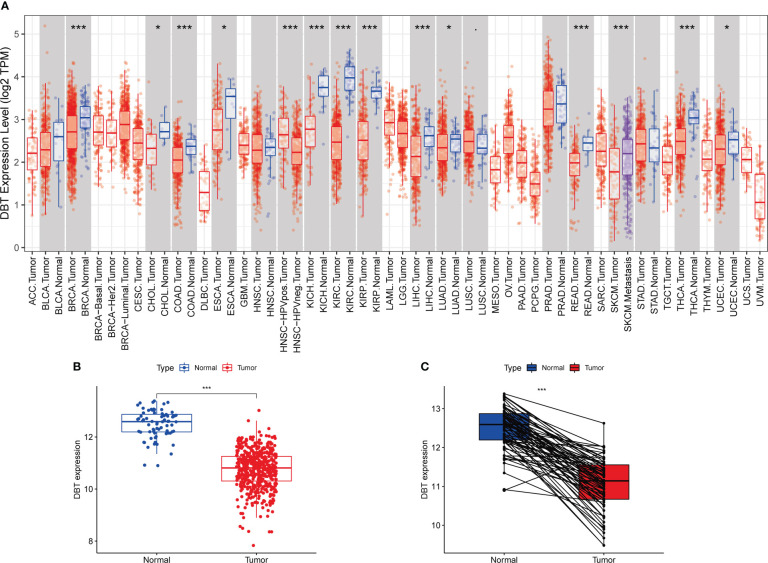
Dihydrolipoamide branched chain transacylase E2 (DBT) expression in patients with kidney renal clear cell carcinoma (KIRC). **(A)** DBT expression in different malignancies as determined by the TIMER database. **(B)** DBT expression is decreased in KIRC tissues as compared to nearby renal tissues. **(C)** DBT expression was determined in 72 matched KIRC and adjacent renal tissues. (*P ≤0.05, ***P ≤ 0.001).

To begin, we analyzed the mRNA expression levels of DBT in various common human malignancies using the TIMER database. Our analysis showed that DBT expression levels were considerably lower in several types of cancers, including KIRC, KIRP, THCA, BRCA, KICH, COAD, LIHC, SKCM, READ, ESCA, CHOL, UCEC, and LUAD tissues, indicating that DBT is likely a tumor suppressor gene (**Figure 1A**). Subsequently, we examined the expression of DBT in KIRC tissues using the TCGA database and found that it was significantly downregulated compared to normal tissues (**Figure 1B**). We further evaluated the expression of DBT in 72 matched KIRC and normal renal samples and observed a reduction in DBT expression in KIRC tissues (**Figure 1C**).

### Association between DBT and clinicopathological features

**Figure 2:**
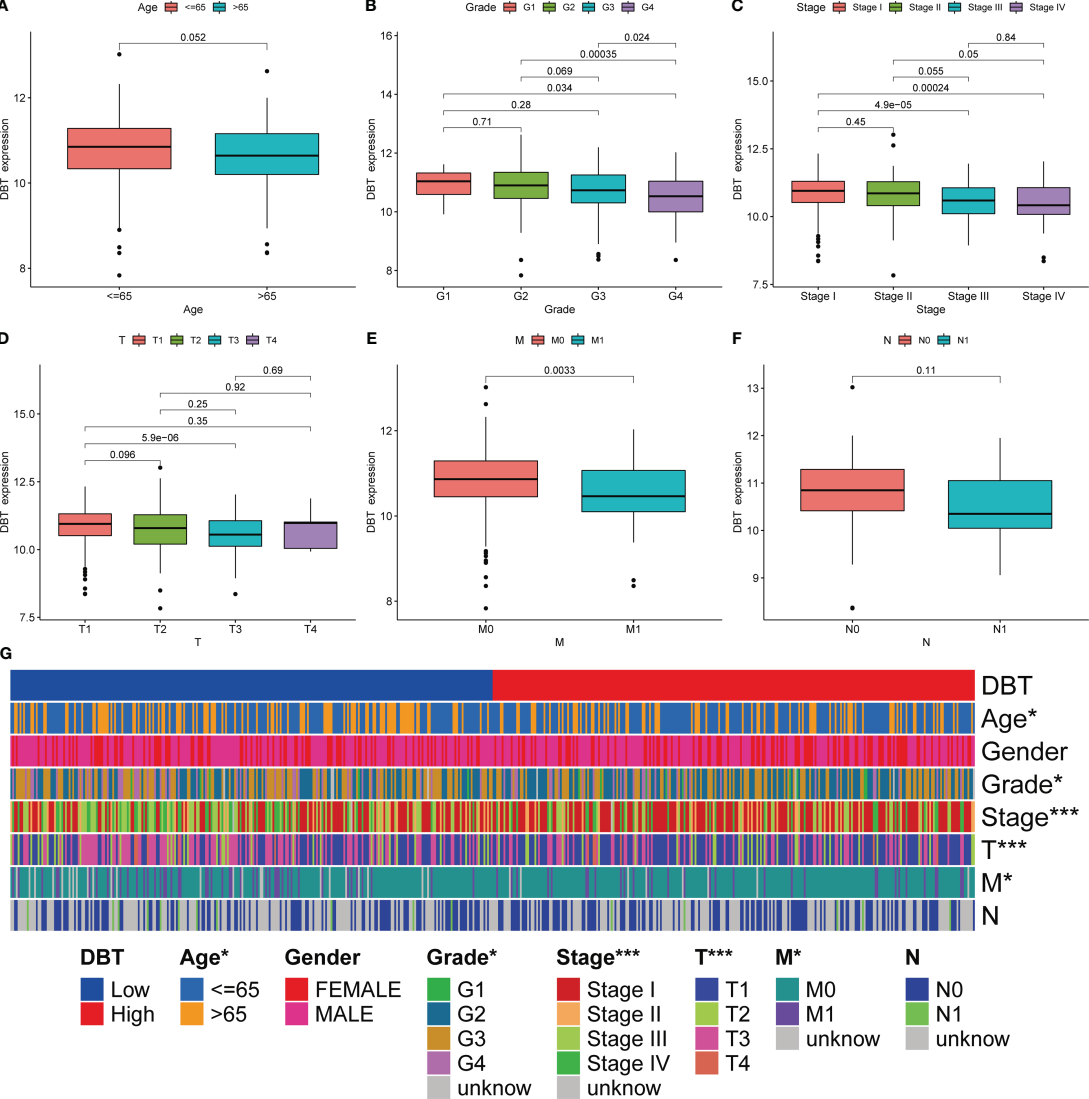
Association of DBT expression with multiple clinicopathological features in KIRC patients. **(A)** Correlation of DBT expression with age in KIRC patients. **(B)** Correlation of DBT expression with grade in KIRC patients. **(C)** Correlation of DBT expression with stage in KIRC patients. **(D)** Correlation of DBT expression with T stage in KIRC patients. **(E)** Correlation of DBT expression with M stage in KIRC patients. **(F)** Correlation of DBT expression with N stage in KIRC patients. **(G)** Correlations between DBT and clinicopathological characteristics are shown in a cluster heatmap. *p<0.05, ***p<0.001.

To further understand the role of DBT in KIRC, we first looked at its link to clinicopathological features. Based on the clinical data from 539 TCGA patients, we discovered that low DBT expression was significantly associated with an elevated WHO grade, stage, T stage, and M stage. DBT expression did not correlate significantly with other clinicopathological factors such as age and N stage (**Figures 2A–F**). The correlation between DBT expression and clinical traits was more vividly shown by the heat map (**Figure 2G**).

### Low DBT expression predicts poor prognosis of KIRC patients

**Figure 3:**
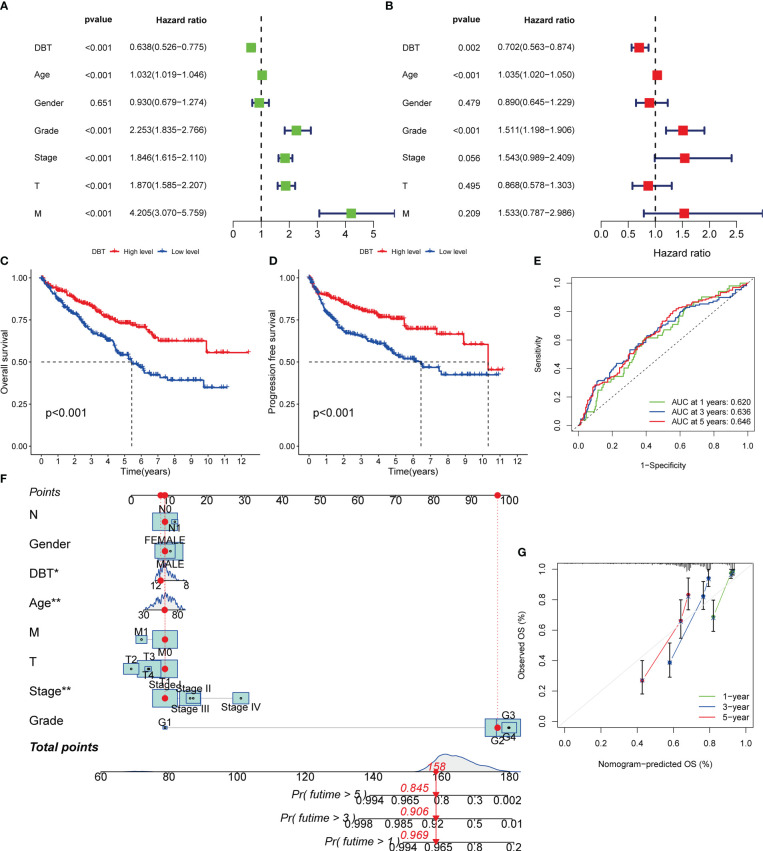
Diagnostic value of DBT expression in KIRC patients. **(A)** Forest plot of DBT expression and clinicopathological features from a univariate Cox regression analysis. **(B)** Forest plot of DBT expression and clinicopathological features from a multivariate Cox regression analysis. **(C)** Overall survival curves for KIRC patients with varying levels of DBT expression. **(D)** Progression free survival curves for KIRC patients with varying levels of DBT expression. **(E)** DBT’s time-dependent ROC curves for predicting 1-, 3-, and 5-year OS in KIRC patients. **(F)** A nomogram for predicting the chance of survival at one, three, and five years in patients with KIRC. **(G)** The nomogram’s calibration curve. *p<0.05, **p<0.01.

We performed univariate Cox regression analysis to identify variables associated with poor prognosis in KIRC patients. The analysis showed that advanced age (HR= 1.032, p < 0.001), increased WHO grade (HR= 2.253, p < 0.001), increased pathological stage (HR= 1.846, p < 0.001), increased T stage (HR= 1.870, p < 0.001), increased M stage (HR= 4.205, p < 0.001), and decreased DBT expression (HR= 0.638, p < 0.001) were all significantly associated with poor prognosis (**Figure 3A**). Subsequently, we performed multiple regression analysis, which showed that WHO grade (HR= 1.511, p < 0.001) and DBT expression (HR= 0.702, p = 0.002) were independent predictive factors in KIRC patients (**Figure 3B**). To further investigate the prognostic significance of DBT expression, we divided KIRC patients into high- and low-DBT expression groups using the appropriate cut-off value. Kaplan-Meier survival analysis showed that patients with low DBT expression had poorer overall survival (OS, p < 0.01, **Figure 3C**) and progression-free survival (PFS, p < 0.001, **Figure 3D**) than those with high DBT expression. Furthermore, receiver operating characteristic (ROC) curves were plotted to assess the predictive value of DBT expression in KIRC patients. The AUCs for DBT in predicting 1-, 3-, and 5-year OS were 0.620, 0.636, and 0.646, respectively, indicating that DBT has a significant predictive value (**Figure 3E**).

A nomogram was employed in this investigation to integrate the important information and predict illness prognosis. Each variable was assigned a number between 1 and 100, as shown in **Figure 3F**. The total point was computed by adding the points for each variable together. The clinical outcomes of KIRC patients were then obtained by drawing a straight line based on the overall point score to validate the patients’ probability of 1-, 3-, and 5-year survival. Additionally, the calibration curve demonstrated that the nomogram’s predicted 3- and 5-year OS rates were very compatible with the observed results (**Figure 3G**). Together, these data established the nomogram’s predictive utility for KIRC patient survival and established the prognostic importance of DBT expression.

### Co-expression of DBT with other genes in KIRC

**Figure 4:**
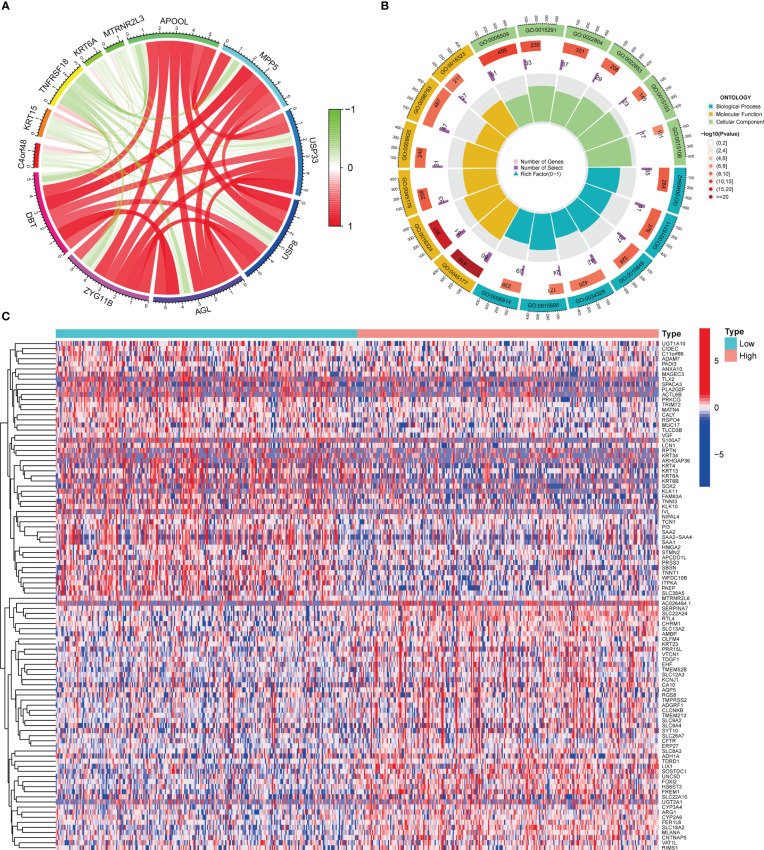
Co-expressed and differentially expressed genes of DBT. **(A)** Circle diagram of co-expressed genes of DBT. **(B)** Circle plot for Gene Ontology (GO) enrichment analysis of differentially expressed genes. **(C)** Heatmap of the top 100 differentially expressed genes.

There were 973 co-expression genes found, 560 of which were favorably associated and 413 of which were negatively connected. We drew a co-expression analysis circle around the 11 most associated genes and discovered that six of them were positively linked, including APOOL, MPP5, USP33, USP8, AGL, and ZYG11B, suggesting that DBT may have a synergistic effect on these genes (**Figure 4A**).

### Functional analyses of DBT-related genes

**Figure 5:**
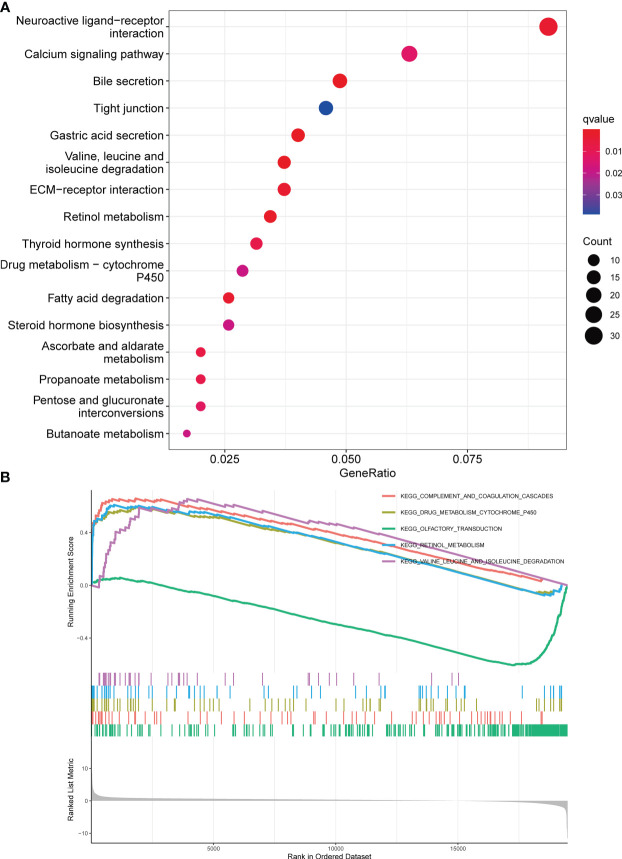
Enrichment analysis of DBT in KIRC. **(A)** Bubble plot for Kyoto Encyclopedia of Genes and Genomes (KEGG) enrichment analysis of differentially expressed genes. **(B)** Gene set enrichment analysis (GSEA) enrichment analysis of differentially expressed genes.

To investigate the involvement of DBT in KIRC, we identified DEGs across subgroups with high and low DBT expression levels using TCGA RNA-seq data. A total of 835 DEGs were identified, comprising 659 upregulated and 176 downregulated genes (**Table S1**). A heat map depicts the top 50 genes that were found to be positively or negatively associated with DBT (**Figure 4C**). GO enrichment analysis showed that DEGs were closely related to transmembrane transport, such as apical part of cell, apical plasma membrane, anion transmembrane transporter activity, secondary active transmembrane transporter activity, and carboxylic acid transport (**Figure 4B**; **Table S2**). KEGG enrichment analysis found that DEGs-related pathways were mainly concentrated in Neuroactive ligand−receptor interaction, Calcium signaling pathway, Bile secretion, Tight junction, and Gastric acid secretion (**Figure 5A**; **Table S3**). To investigate the putative molecular activities of DBT in KIRC, GSEA was used to predict DBT-related signaling pathways between DBT-low and DBT-high tumor samples (**Figure 5B**). Immunotherapeutic and drug metabolism-related pathways, such as complement and coagulation cascades, and drug metabolism cytochrome p450, were enriched in the high DBT group. Additionally, our GSEA analysis revealed that DBT is connected with a number of cancer-related signaling pathways not shown in the image, including the PPAR signaling pathway, the TGF signaling pathway, the KEGG-pancreatic cancer, the KEGG-prostate cancer, and the KEGG-renal cell cancer. Taken together, our findings imply that DBT may be a potential immunologic marker in KIRC (**Table S4**).

### Correlation between DBT expression and tumor microenvironment

**Figure 6:**
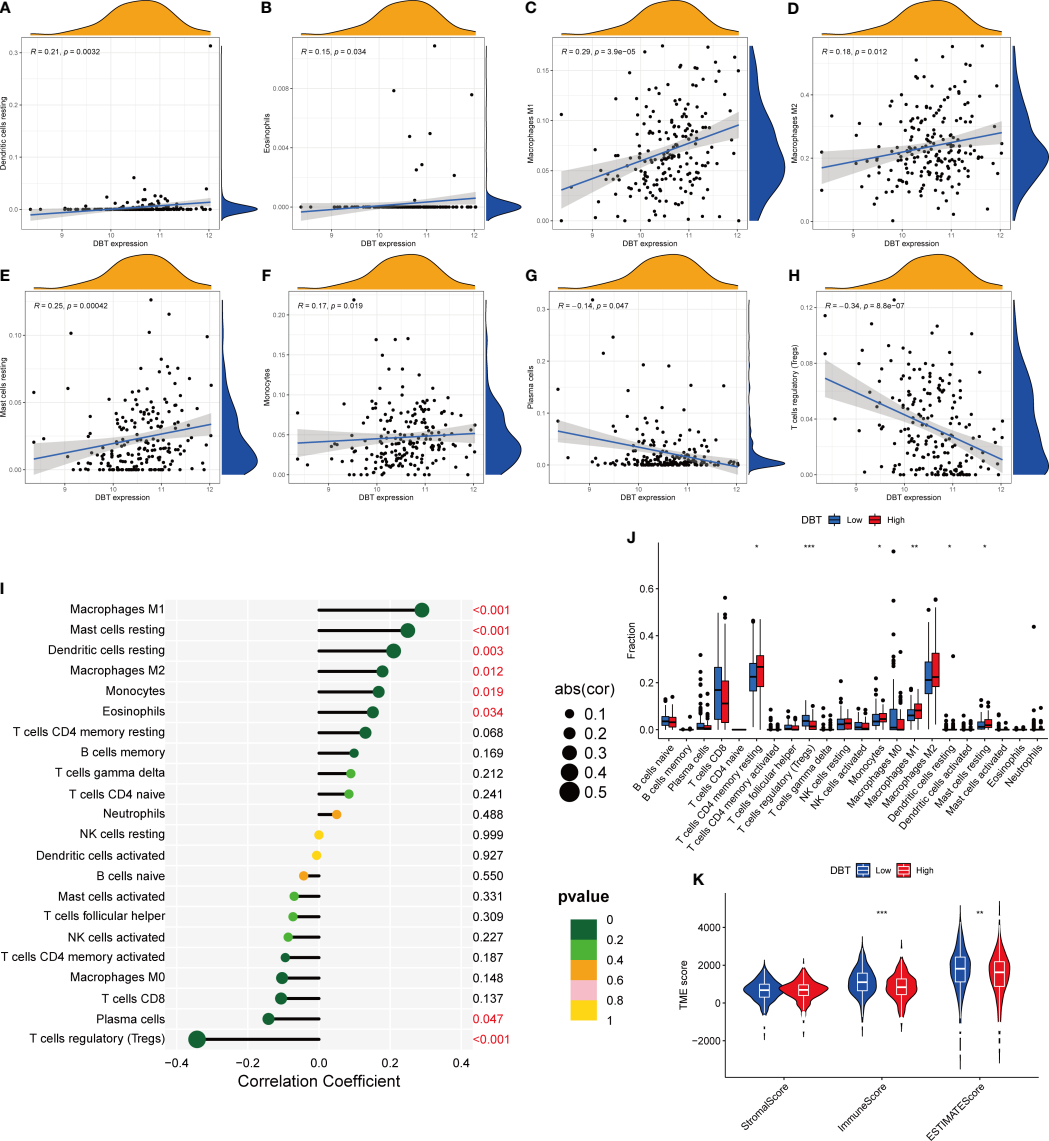
Correlation between DBT expression and tumor microenvironment. **(A-H)** The scatter plot depicts the correlation between tumor-infiltrating immune cells and DBT expression. **(I)** The forest plot demonstrating the correlations between DBT expression and the 22 tumor-infiltrating immune cells. **(J)** Ratio differences of 22 immune cells relative to DBT expression levels in KIRC tumor samples. **(K)** Violin plot showing the association between tumor microenvironment (TME) scores and DBT expression levels. *p<0.05, **p<0.01, ***p<0.001.

**Figure 7:**
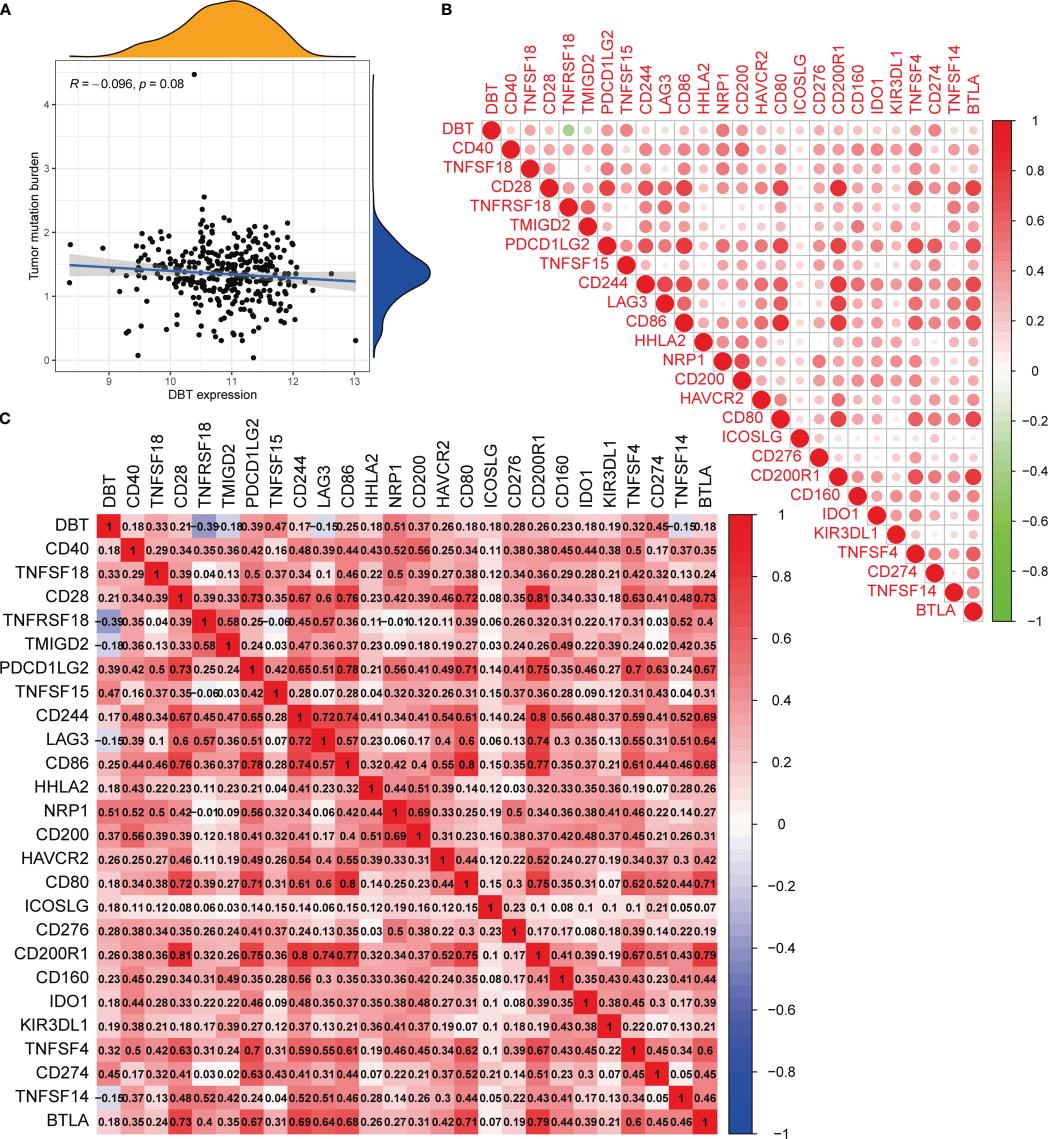
Analysis of the tumor mutation burden and immunological checkpoints. **(A)** The correlation between tumor mutation burden (TMB) and DBT expression. There is no statistical significance indicated in this case. **(B, C)** Heatmap showing correlations between DBT and 25 immune checkpoints. Correlation coefficients are shown by the numbers in each little box. The color red shows a positive association between two genes, whereas the color blue suggests the inverse. The deeper the shade, the stronger the association.

The CIBERSORT approach was used to validate the link between DBT expression and the immunological component by creating 22 distinct immune cell profiles and assessing the fraction of tumor-infiltrating immune subtypes in KIRC patients. Eight distinct classes of tumor-infiltrating immune cells (TICs) were discovered to be associated with DBT expression (P<0.001, **Figures 6A–H**). The findings indicated that six TICs, including M1 macrophages, mast cells, resting dendritic cells, M2 macrophages, monocytes, and eosinophils correlated positively with DBT expression, but two types of TICs, comprising plasma cells, and regulatory T cells (Tregs), correlated negatively with DBT expression (**Figures 6I, J**). We calculated immune scores, stromal scores, and ESTIMATE scores in different subgroups, and we found that immune score and ESTIMATE scores were higher in the DBT low-expression group, while stromal scores had no statistical significance with their expression (**Figure 6K**). The data above show that DBT may contribute to the immunological response in the TME through its effect on immune cells. Increased immune cell infiltration may result in a stronger anti-tumor impact, which may possibly explain why patients with high DBT expression had a favorable prognosis. Additionally, no significant variations in tumor mutation burden (TMB) were identified between the groups with high and low DBT expression (**Figure 7A**).

### DBT expression as a prognostic factor in tumor immunotherapy

Immune checkpoints are a group of molecules produced on immune cells that govern the extent to which the immune system is activated. They are critical in avoiding the onset of autoimmune disease. To begin, we discovered that most immunological checkpoints were positively linked with DBT expression, except for four immune checkpoints, TNFRSF18, TMIGD2, TNFSF14, and LAG3 (**Figure 7B**). Notably, we found that PDCD1LG2 (PD-L2), CD274 (PD-L1), and CTLA-4 (CD28, CD80, CD86), which are immune checkpoints, showed positive correlation with DBT expression, respectively (**Figure 7C**).

**Figure 8:**
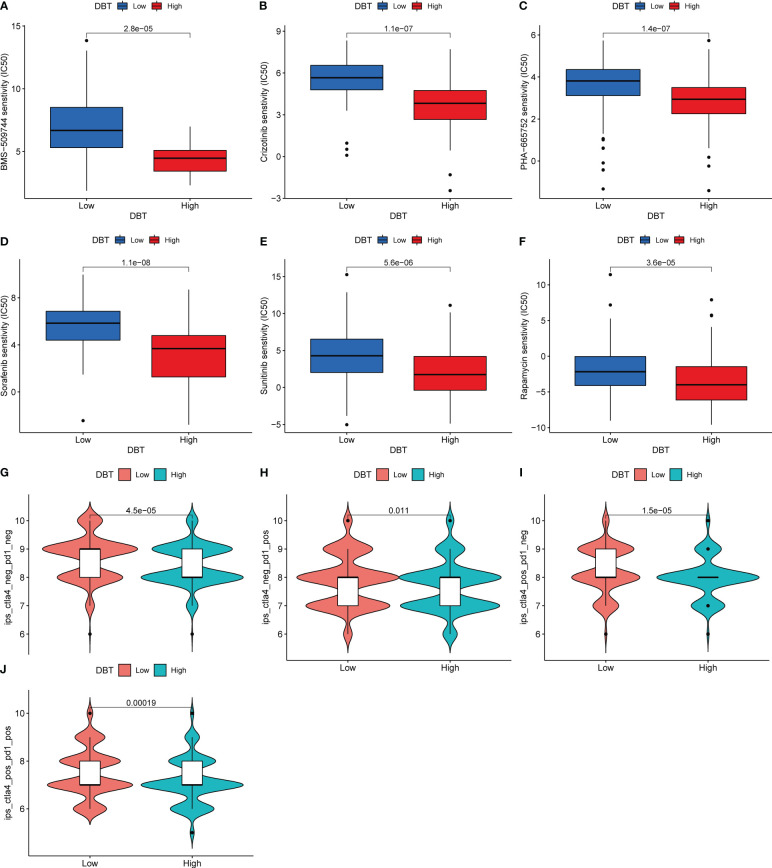
DBT expression as a prognostic factor in tumor immunotherapy. **(A–F)** Drug sensitivity analysis between two DBT expression subgroups. **(G–J)** Immunotherapy response between two DBT-expressing subgroups.

We next assessed the sensitivity of KIRC patients with different DBT expression levels to various targeted agents (**Figures 8A–F**). Patients with high DBT expression showed reduced IC50 values for chemotherapeutic medicines such as BMS-509744 (ITK inhibitor), crizotinib (ALK inhibitor), PHA-665752 (MET inhibitor), rapamycin (mTOR inhibitors), sorafenib (multiple kinase inhibitor), and sunitinib (tyrosine kinase inhibitor). As a result, we speculate that patients with elevated DBT expression may be more responsive to and efficacious with the aforementioned medicines. Following that, we assessed patients’ IPS in distinct DBT expression groups using the KIRC-IPS cohort. The results indicated that there were statistically significant variations in IPS between patients with high and low DBT expression (**Figures 8G–J**). In KIRC patients receiving both anti-PD-1 and anti-CTLA4 therapy, the relationship between IPS values and DBT expression was inverse, suggesting a greater therapeutic effect in individuals with low DBT expression. Additionally, similar outcomes were achieved when patients were treated with anti-CTLA4 or anti-PD-1 antibodies. This further demonstrated that patients with reduced DBT expression were more amenable to immunotherapy.

### DBT inhibits KIRC cell proliferation, migration, and invasion

**Figure 9:**
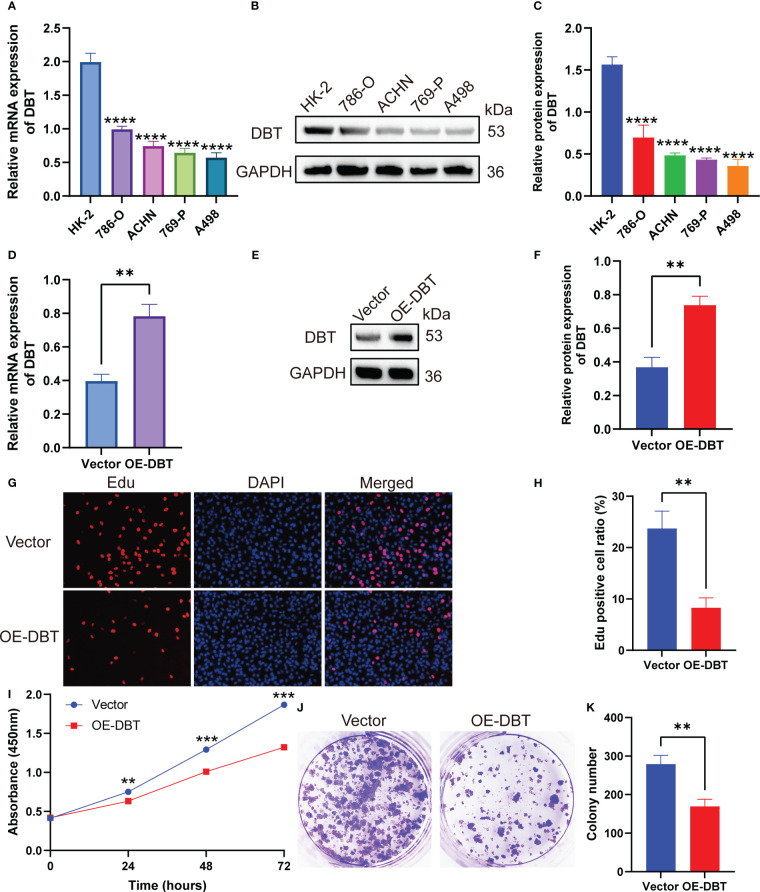
DBT inhibits the proliferation of KIRC cells. **(A)** RT-qPCR analysis showed that DBT mRNA expression was significantly lower in the KIRC cell line than in the normal kidney tissue cell line. **(B, C)** Protein blot analysis showed that DBT protein expression was significantly lower in the KIRC cell line than in the normal renal tissue cell line. **(D–F)** Plasmid transfection efficiency in A498 cells was verified by RT-qPCR and western blotting. **(G, H)** The number of A498 cells that stained positive for 5-ethynyl-2′-deoxyuridine (EdU) was much lower in the OE-DBT group, which shows that their growth was slowed down. **(I)** The Cell Counting Kit-8 (CCK-8) assay showed significantly lower cell activity in the OE-DBT group than in the Vector group at 24, 48, and 72 hours. **(J, K)** The number of colonies formed was significantly lower in the Vector group than in the OE-DBT group in A498 cells. **p<0.01, ***p<0.001, ****p<0.0001. Vector: control overexpression plasmid; OE-DBT, DBT overexpression plasmid.

**Figure 10:**
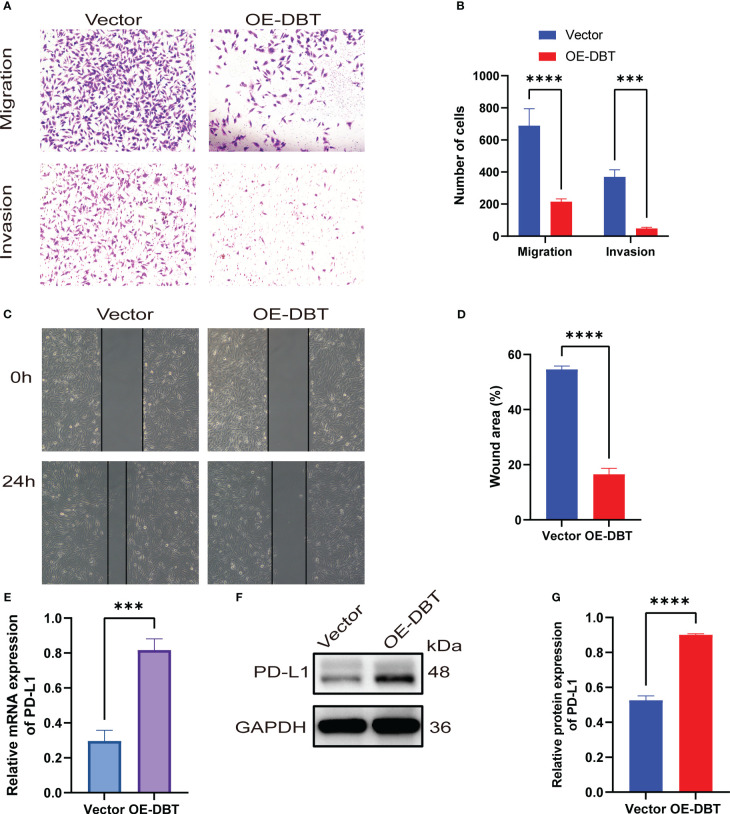
KIRC cells’ migration and invasion are inhibited by DBT. **(A, B)** Transwell experiments with or without Matrigel were used to evaluate the migration and invasion capabilities of A498 cells expressing DBT. **(C, D)** Evaluation of A498 cell migration ability using wound healing experiments. **(E)** The mRNA expression level of PD-L1 was significantly increased after overexpression of DBT in A498 cell lines. **(F, G)** The protein expression level of PD-L1 was also significantly increased after overexpression of DBT in A498 cell lines. The error bars on the histogram represent the standard deviation (SD) of measurements that were performed in triplicate. ***p<0.001, ****p<0.0001. Vector: control overexpression plasmid; OE-DBT, DBT overexpression plasmid.

To investigate the biological activities of DBT in KIRC cell processes, we compared the mRNA and protein levels of DBT in four KIRC cell lines (786-O, ACHN, 769-P, and A498) and a normal kidney tissue cell line (HK2) using RT-qPCR and western blotting. The findings suggested that when compared to the normal renal cell line, the four KIRC cell lines had significantly lower levels of DBT mRNA (**Figure 9A**) and protein expression (**Figures 9B, C**). A498 cell line was transfected with the overexpression plasmid (OE-DBT) and the control plasmid (Vector) to overexpress DBT due to its relatively low expression. RT-qPCR (**Figure 9D**) and western blotting (**Figures 9E, F**) both validated the transfection effectiveness. EdU test findings demonstrated that the overexpression of DBT considerably decreased the proliferative potential of KIRC cells relative to controls (**Figures 9G, H**). CCK-8 assay indicated that DBT overexpression decreased A498 cell growth significantly (**Figure 9I**). The colony formation experiment revealed that the OE-DBT groups had significantly fewer colonies than the Vector groups (**Figures 9J, K**). These findings suggested that DBT may inhibit KIRC cell growth as a tumor suppressor. Using transwell assays, the effects of DBT on the migratory and invasion capabilities of KIRC cells were investigated further. Overexpression of DBT dramatically reduced the amount of migrating and invading cells, according to the findings (**Figures 10A, B**). Then, we conducted wound healing assays. The results demonstrated that after 24 hours of scratching, the migration rate of OE-DBE cells was considerably lower than that of the control KIRC cells (**Figures 10C, D**).

Previous bioinformatics analysis has suggested a positive correlation between DBT and PD-L1, as well as a possible reciprocal regulation between DBT and PD-L1. To investigate this further, we examined the expression levels of PD-L1 in A498 cell lines that overexpressed DBT. We found that both the mRNA (**Figure 10E**) and protein (**Figures 10F, G**) expression levels of PD-L1 were significantly higher in the OE-DBT group compared to the Vector group. Our results indicate that DBT positively regulates the expression of PD-L1, thereby potentially impacting the tumor microenvironment.

## Discussion

KIRC is the most common and deadly urological malignancy. Localized KIRC can be cured through nephrectomy, radio-ablation, and active surveillance, while metastatic renal cell carcinoma requires a combination of surgery and systemic medication (19). Although conventional treatment has a poor response rate, a number of innovative therapeutics for metastatic renal cell carcinoma have emerged lately, including targeted medicines and novel immunotherapeutic drugs (20). Nonetheless, resistance development and limited sustained responses need the development of new anticancer agents with increased selectivity and effectiveness (21). Leucine, isoleucine, and valine, which are key BCAAs involved in the regulation of protein synthesis, are receiving considerable attention for their significance in regulating glucose homeostasis, as well as their critical role in activating the mTOR signaling pathway (22). There is evidence that the catabolism of BCAA plays a critical role in the pathogenesis of many diseases, including diabetes, heart failure, Alzheimer’s disease, cirrhosis, and cancer (23). Furthermore, the correction of dysregulated BCAA metabolism may improve disease prognosis and prevent serious complications (24).

In this study, we discovered that DBT expression was down-regulated in various human malignancies, including KIRC. We observed that low DBT expression was associated with more severe clinicopathological characteristics and a poor prognosis for KIRC patients. We found DBT as an independent prognostic factor in KIRC by multifactorial Cox analysis and therefore created a nomogram including DBT to predict the survival time of patients. We also identified genes co-expressed with DBT, most of which were positively correlated with DBT and warranted further investigation of their relationship. Following that, we identified DBT-associated DEGs using bioinformatics analysis and discovered that DBT regulates a number of cancer-related signaling pathways, including the PPAR signaling route, TGF signaling pathway, KEGG-pancreatic cancer, KEGG-prostate cancer, and KEGG-renal cell carcinoma. DBT expression has also been linked to immune microenvironment in KIRC, making it useful for identifying immune checkpoints, choosing immunotherapy, and predicting medication susceptibility. Our findings suggest that DBT may have predictive significance and play a role in the development and progression of KIRC tumors.

In our study, we observed a positive correlation between DBT expression and six types of tumor-infiltrating immune cells (TICs): M1 macrophages, mast cells, resting dendritic cells, M2 macrophages, monocytes, and eosinophils. However, we found a negative correlation between DBT expression and two types of TICs, namely plasma cells and regulatory T cells (Tregs). According to general beliefs, M1 macrophages are anti-tumor cells, whereas M2 macrophages support tumor growth. The number of macrophages is substantially linked with poor prognosis in several kinds of human malignancies, especially those with M2 polarization (25–28). While TAMs are not identical to M1 or M2, they are often M2-like and promote tumor development by inhibiting the immune system. It has been shown that macrophage activation and function are regulated by a variety of amino acids, among which BCAAs contribute to the macrophage activation state, which may explain why DBT and macrophages are so closely linked in KIRC. Specifically, BCAAs like leucine provide acetoacetate, acetyl coenzyme A, and glutamine, which can enter the TCA cycle to influence metabolism and thus stimulate macrophage activation (29). Medical research has shown that BCAT1, the primary isoform of branched-chain aminotransferase in human macrophages, has an impact on oxygen consumption, glycolysis, immune response gene expression, and itaconic acid production, which supports our hypothesis (30). Tregs are required for the maintenance of homeostasis and immunological tolerance *in vivo*; hence, mature Tregs block the immune response, thereby promoting tumor development (31). Several studies have shown that Foxp3 plays an important role in the formation and function of these cells, and current research has underlined Foxp3’s involvement in epigenetic control of Tregs (32, 33). Mast cells are thought to be major coordinators of anti-tumor immunity and are involved in the pathophysiology of allergy and autoimmune disorders (34). As a result, mast cells constitute an underutilized yet very potential target for tumor immunotherapy (35). Additionally, research into upstream drivers of T cell activation, such as antigen-presenting dendritic cells that launch the tumor immune cycle with T cells, is rising (36). In conclusion, our findings suggest that DBT in KIRC functions as a cancer suppressor by activating M1 macrophages, mast cells, and dendritic cells while suppressing regulatory T cells, which promotes anti-cancer immune responses.

Notably, we found that PDCD1LG2 (PD-L2), CD274 (PD-L1), and CTLA-4 (CD28, CD80, CD86), which are immune checkpoints, showed positive correlation with DBT expression, respectively. We investigated the expression levels of PD-L1 in A498 cell lines that overexpressed DBT and observed higher levels of mRNA and protein expression of PD-L1 in the OE-DBT group compared to the Vector group. These findings suggest that DBT may play a role in the tumor microenvironment and potentially impact the effectiveness of immunotherapy. However, the exact mechanism underlying this phenomenon requires further investigation. Following that, we assessed patients’ IPS in distinct DBT expression groups using the KIRC-IPS cohort. In KIRC patients receiving both anti-PD-1 and anti-CTLA4 therapy, the relationship between IPS values and DBT expression was inverse, suggesting a greater therapeutic effect in individuals with low DBT expression. Additionally, similar outcomes were achieved when patients were treated with anti-CTLA4 or anti-PD-1 antibodies only. The combination of ipilimumab and nivolumab has shown further improvements in clinical outcomes and has been authorized for first-line therapy of patients with moderate to poor risk metastatic renal cell carcinoma (37). Long before the advent of contemporary ICIs, KIRC was recognized as a tumor form that was known to respond to immune-based therapy (38). Previously, KIRC was treated with cytokine-based immunotherapy, particularly high dosage IL-2, which resulted in a limited proportion of patients achieving lasting full responses (39). Despite the fact that ICI-based combinations have drastically improved outcomes for patients with advanced KIRC, the majority of patients still exhibit primary resistance to these drugs or develop resistance after an initial response (40, 41). For patients with this condition, the discovery of innovative treatment techniques aimed at overcoming these mechanisms of resistance is critical (42).

We investigated the responsiveness of KIRC patients with varying levels of DBT expression to different targeted agents. Patients with high DBT expression showed lower IC50 values for chemotherapeutic agents such as BMS-509744 (an ITK inhibitor), crizotinib (an ALK inhibitor), PHA-665752 (a MET inhibitor), rapamycin (an mTOR inhibitor), sorafenib (a multiple kinase inhibitor), and sunitinib (a tyrosine kinase inhibitor). As a result, we speculate that patients with elevated DBT expression may be more responsive to and efficacious with the aforementioned medicines. A recent study found that patients receiving first-line nivolumab and cabozantinib had a better progression-free survival than those getting sunitinib (43). MET mutations have been found to be potential drivers of tumor development in RCC and hence a promising therapeutic target (44). By blocking VEGF resistance pathways, combination treatments of VEGF and MET inhibitors may result in prolonged and profound responses even in non-MET driven KIRC (45). Additionally, the addition of checkpoint inhibitors to MET inhibition has indicated early effectiveness (46). The role of the mechanistic target of rapamycin (mTOR) in the biology of KIRC is highlighted by many factors (47). The mTOR signaling pathway is typically active in KIRC and promotes cancer cell proliferation and survival (48). mTOR also increases tumor angiogenesis and controls the production of hypoxia-inducible proteins, both of which are significant in KIRC (49, 50). Despite their protumorigenic effects, mTOR inhibitors have failed to give long-term anticancer advantages in KIRC patients, indicating that their function in the treatment of KIRC should be reconsidered (51).

## Conclusion

In conclusion, our research revealed that DBT expression was significantly reduced in KIRC and that its low expression was linked with disease progression and poor patient survival. Experiments revealed that KIRC cell growth, migration, and invasion were decreased by DBT overexpression. In addition, the method of action of DBT in the tumor immunological milieu was revealed. Our data indicated that DBT may cause cancer by triggering abnormal inflammatory and immune responses. This research contributes to our knowledge of KIRC’s pathophysiology and molecular targets.

## Data availability statement

The original contributions presented in the study are included in the article/**Supplementary Material**. Further inquiries can be directed to the corresponding authors.

## Author contributions

XX and HH designed the study. CZ, JY, YJ, YH, and ZY analyzed and interpreted the data. RH collected recent advances and references. CZ summarized the data and wrote the manuscript. XX checked and revised the manuscript. All authors contributed to the article and approved the submitted version.
